# Hospitalization Outcomes Related to Acute Kidney Injury in Inpatients With Acute Myocardial Infarction: A Cross-Sectional Nationwide Study

**DOI:** 10.7759/cureus.26490

**Published:** 2022-07-01

**Authors:** Gagan Kaur, Rushi P Shah, Aabha Shakya, Chia Chi Loh, Sravani Kommuru, Syed Nurul Aziz, Viralkumar Patel

**Affiliations:** 1 Medicine and Surgery, Sri Guru Ram Das Institute of Medical Sciences and Research, Amritsar, IND; 2 Medicine, Byramjee Jeejeebhoy Medical College, Ahmedabad, IND; 3 Family Medicine, Saint James School of Medicine, Kingstown, VCT; 4 Internal Medicine, Manipal University College Malaysia, Melaka, MYS; 5 Internal Medicine, Dr. Pinnamaneni Siddhartha Institute of Medical Sciences & Research Foundation, Vijayawada, IND; 6 Internal Medicine, Shaheed Suhrawardy Medical College, Dhaka, BGD; 7 Internal Medicine, Sarasota Memorial Health Care System, Sarasota, USA

**Keywords:** in-hospital outcome, risk factors‎, cardiogenic shock, acute myocardial infarction, acute kidney injury

## Abstract

Objective

To delineate the differences in demographic characteristics and hospitalization outcomes in patients with acute myocardial infarction by comorbid acute kidney injury (AKI) and to explore the risk factors for in-hospital mortality due to AKI in acute myocardial infarction (AMI) inpatients.

Methods

We conducted a retrospective cross-sectional study using a nationwide inpatient sample and included 77,585 adult inpatients with AMI and further divided by the presence of a co-diagnosis of AKI. A logistic regression model was used to evaluate the odds ratio (OR) of the association between in-hospital mortality and AKI and other comorbidities.

Results

The prevalence of AKI in AMI inpatients during hospitalization was 11.69%. Among AMI inpatients with AKI, it was prevalent in males (73.9%) and whites (48.8%). Patients with AKI had a higher prevalence of complicated comorbid hypertension (58.7%), diabetes with complications (34.8%), cardiogenic shock (17.4%), and drug abuse (12.3%). Male patients had lower odds of in-hospital mortality (OR 0.69; 95% Cl 0.61-0.79) compared to females. Hispanics had a higher association with mortality (OR 1.45; 95% Cl 1.21-1.74) than whites and other races/ethnicities. Patients who developed cardiogenic shock were at 17 times higher odds of in-hospital mortality (OR 17.25; 95% CI 15.14-19.67), followed by AKI (OR 4.64; 95% CI 4.06-5.31), and alcohol abuse (OR 1.29; 95% CI 1.03-1.64). The in-hospital mortality rate among AMI inpatients with AKI (7.6%) was significantly higher compared to that seen in the non-AKI cohort (0.9%).

Conclusion

AMI inpatients with AKI during hospitalization was prevalent in males and whites. Among the demographic risk factors, females and Hispanics had a higher likelihood of in-hospital mortality during the inpatient management of AMI. Cardiogenic shock and AKI increased the odds of in-hospital mortality compared to other comorbidities in AMI inpatients.

## Introduction

Acute kidney injury (AKI) encompasses a broad spectrum of renal pathology. AKI is defined as an increase in serum creatinine of ≥0.3 mg/dL within 48 hours or ≥50% within seven days as well as urine output of <0.5 mL/kg/hour for less than six hours [1]. AKI is known to be triggered by many medical and surgical conditions and increases mortality, length of stay (LOS), and costs in hospitalized patients [2, 3]. It is estimated that AKI occurs in 20-2000 per million population, of which 7% to 18% in hospitalized patients and around 50% of patients are in intensive care units [4, 5]. Roughly two million people die worldwide due to AKI, leading to having high morbidity and mortality rate. Hospitalized patients with AKI may require hemodialysis, which increases the mortality risk. Those who survive the condition have a higher risk of developing chronic kidney disease (CKD) and end-stage renal disease, which carries a high economic, societal and personal burden [6, 7].

Some known risk factors for developing AKI include older age, preexisting hypertension, preexisting CKD, anterior myocardial infarction, and furosemide use among others [3]. Among these, acute myocardial infarction (AMI) is a critical risk factor and known to increase the risk of AKI in hospitalized patients [3]. Wang et al. established the incidence of AKI in AMI patients to be as high as 26% [3]. When examined with the high frequency of AMI on its own, where one person suffers an AMI every 40 seconds, this suggests a high burden of the disease [8]. However, there has been a significant change in AMI mortality data over the last decade. Based on the heart and disease and stroke update, the 30-day mortality among hospitalized patients with AMI has been declining based on the data from Medicare. The mortality rate due to AMI decreased from 20% in 1995 to 12.4% in 2014 [9]. Nevertheless, causes and long-term risks for mortality in AMI patients have not been extensively studied [10]. The existence of comorbid conditions, a decrease in renal perfusion in AMI patients causes significant kidney injury in hospitalized patients. Patients with AKI are at higher risk of developing recurrent AMI, heart failure, and CKD leading to long-term mortality [5].

Thus, we aim to delineate the differences in demographic characteristics and hospitalization outcomes, including the severity of illness, disposition status and in-hospital mortality, and hospitalization stay and cost in AMI inpatients by comorbid AKI. Also, we aim to explore the risk factors for in-hospital mortality due to AKI and cardiogenic shock in AMI inpatients.

## Materials and methods

Study sample

We conducted a retrospective cross-sectional study using the nationwide inpatient sample (NIS) from Jan 1, 2019, to Dec 31, 2019 [11]. The NIS is the largest inpatient database representing non-federal community hospitals from 48 states plus the District of Columbia in the United States. According to the agency for healthcare research and quality (AHRQ) and the department of health and human services, as the NIS is a secured de-identified database, it does not require approval from an institutional review board [11]. We included 77,585 adult inpatients (age 18-50 years) hospitalized on emergency-based admissions with a primary diagnosis of acute myocardial infarction (AMI). The study sample was divided by the presence of a comorbid diagnosis of acute kidney injury (AKI) [11].

Variables

The variable of interest included demographic characteristics (age, sex, and race) and comorbidities which are the co-diagnoses in the patient records. We included alcohol abuse, complicated diabetes, hypertension (complicated), drug abuse, and cardiogenic shock. The hospitalization outcomes of interest included: severity of illness, length of stay (LOS), total charges, and in-hospital mortality (all-cause). The severity of illness was evaluated using the all-patient refined drugs (APR-DRGs), which was developed by 3M Health Information Systems and stratified as minor, moderate, and major loss of functioning [11]. LOS was calculated by subtracting the admission date from the discharge date, and the total charges do not include professional fees and non-covered charges [11].

Statistical analysis

We used Pearson’s chi-square test and independent-sample t-test for categorical and continuous data, respectively. Descriptive statistics were used to summarize the differences between AKI and non-AKI groups. The mean and standard deviations were used to explain the constant variables, including age, LOS, and total charges. A binomial logistic regression model was used to evaluate the odds ratio (OR) of the association between in-hospital mortality and AKI and other comorbidities. A P-value <0.01 was used as a reference to determine the statistical significance test result. All statistical analysis was done using the statistical package for the social sciences (SPSS) version 27 (IBM Corp., Armonk, NY).

## Results

We included 77,585 adults with AMI, and the majority of the study inpatients were males (69.4%) and whites (60.7%). Also, 30.6% study sample consisted of women. The most common comorbidities among the AMI inpatients were complicated hypertension (27.6%), followed by complicated diabetes (20.1%), drug abuse (8.3%), alcohol abuse (5.3%), and cardiogenic shock (4.5%).

AKI prevalence in AMI inpatients during hospitalization was 11.69% (N = 9,070). There was no statistically significant difference between AKI and non-AKI cohorts by age (P = 0.063). Among AMI inpatients who had an episode of AKI, it was more prevalent in males (73.9%) and whites (48.8%), and when compared to the non-AKI cohort, the blacks had a higher prevalence of an AKI episode (29.7% vs 17.8%).

There was a higher prevalence of comorbidities in the AKI cohort, including hypertension with complications (58.7% vs 23.4%), diabetes with complications (34.8% vs 18.1%), cardiogenic shock (17.4% vs 2.7%), drug abuse (12.3% vs 7.7%) and alcohol abuse (6.3% vs 5.2%) compared to the non-AKI cohort and it was statistically significant.

The severity of illness was higher in the AKI cohort, with a major loss of functioning (62.6%) compared to that seen in non-AKI (17.8%). AMI inpatients with AKI had a higher mean LOS (6.8 days vs 2.9 days) and higher mean total charges ($173,193 vs $87,657) compared to the non-AKI cohort. A significant difference was in in-hospital mortality among AMI inpatients with AKI (7.6%) compared to that seen in the non-AKI cohort (0.9%), as shown in Table 1.

**Table 1:** Differences in demographics and hospital outcomes in acute myocardial infarction inpatients AKI: Acute kidney injury; LOS: Length of stay

Variable	AKI (no)	AKI (yes)	Total	P-value
Number of inpatients	68515	9070	77585	-
Mean age at admission, in years	43.7	43.9	-	0.063
Sex, in %				
Male	68.8	73.9	69.4	<0.001
Female	31.2	26.1	30.6	
Race/ethnicity, in %				
White	62.2	48.8	60.7	<0.001
African American	17.8	29.7	19.2	
Hispanic	11.8	13.4	12.0	
Other	8.2	8.1	8.2	
Comorbidities, in %				
Alcohol abuse	5.2	6.3	5.3	<0.001
Diabetes with complications	18.1	34.8	20.1	<0.001
Hypertension, complicated	23.4	58.7	27.6	<0.001
Cardiogenic shock	2.7	17.4	4.5	<0.001
Drug abuse	7.7	12.3	8.3	<0.001
Severity of illness, in %				
Minor loss of function	47.4	0	41.8	<0.001
Moderate loss of function	34.9	37.4	35.2	
Major loss of function	17.8	62.6	23.0	
Other outcomes				
Mean LOS, in days	2.9	6.8	-	<0.001
Mean total charges, in $	87,657	173,193	-	<0.001
In-hospital mortality, in %	0.9	7.6	1.7	<0.001

Even though the prevalence of AMI among males was comparatively high, they had lower odds of in-hospital mortality (OR 0.69; 95% CI 0.61-.79) compared to the females. Compared to the whites, only Hispanics had a higher statistically significant association with in-hospital mortality (OR 1.45; 95% CI 1.21-1.74). There existed a statistically significant association between certain comorbidities and in-hospital mortality. Patients who developed cardiogenic shock were at 17 times higher odds of in-hospital mortality (OR 17.25; 95% CI 15.14-19.67), followed by AKI (OR 4.64; 95% CI 4.06-5.31), and alcohol abuse (OR 1.29; 95% CI 1.03-1.64) shown in Table 2.

**Table 2:** Risk factors for in-hospital mortality in acute myocardial infarction inpatients

Variable	Odds ratio	95% Confidence interval		P-value
		Lower limit	Upper limit	
Mean age at admission	1.03	1.02	1.04	<0.001
Sex				
Female	Reference			
Male	0.69	0.61	0.79	<0.001
Race/ethnicity				
White	Reference			
African American	1.11	0.95	1.30	0.203
Hispanic	1.45	1.21	1.74	<0.001
Other	0.94	0.75	1.18	0.600
Comorbidities				
None	Reference			
Alcohol abuse	1.29	1.03	1.64	0.027
Diabetes with complications	1.05	0.90	1.21	0.551
Hypertension, complicated	0.73	0.63	0.83	<0.001
Cardiogenic shock	17.25	15.14	19.67	<0.001
Drug abuse	0.41	0.31	0.54	0.161
Acute kidney injury	4.64	4.06	5.31	<0.001

## Discussion

The prevalence of AKI was 11.69% in AMI inpatients among 77,585 adults in our sample. Until recently, the lack of uniformity in AKI definition has led to a wide range of variations in its prevalence data. In the subset of patients with AMI-induced AKI, the prevalence reported worldwide ranges from 15.8% to 55% [12]. A similar study from China reported AKI incidence in hospitalized AMI adults at 26%, a relatively high number when we look at their sample size of 1,124 patients. Additionally, they reported a mortality rate in the AKI group to be 20%, whereas our cohort had an inpatient mortality rate of 7.6% [3].

Wang et al. studied AKI and mortality in 19,249 hospitalized patients reporting a 22.7% AKI incidence, of which black and older patients were at higher risk of developing AKI [13]. The same cohort reported the most common primary discharge diagnosis as circulatory diseases (25.4%), including ischemic heart disease such as AMI [13]. We found a similar trend of higher incidence of AKI among African American and older inpatients with AMI in our dataset. We suspect this is explained by the higher risk of AKI among all African American patients compared to whites [14]. African Americans are four times more likely to suffer from AKI than whites [15]. Diabetes and hypertension are the most common cause of AKI in African American population [15]. Our data also found that Hispanics were at 1.5 times higher risk for inpatient mortality than whites which were supported by a study done by Kim et al. [16].

In addition to high AKI prevalence in AMI inpatients, we also analyzed the association with some severe comorbidities. In line with previous studies, we found a high prevalence of complicated hypertension and diabetes in the AKI cohort compared to the non-AKI cohort among AMI inpatients. One of the microvasculature complication of diabetes mellitus is diabetic nephropathy [17]. Also, diabetes is known to be the leading cause of end-stage renal disease in the US [17]. Studies have proven diabetes to be an independent risk factor for AKI [17].

AMI patients with cardiogenic shock or those undergoing coronary artery bypass grafting (CABG) are another subset of patients at higher risk of AKI [18, 19]. Conversely, we also found a higher prevalence of cardiogenic shock (17.4%) in the AKI cohort compared to the non-AKI cohort in AMI inpatients. The cardiogenic shock causes end-organ hypo perfusion, which may result in AKI, and prompt treatment for both conditions is crucial to avoid irreversible organ damage. Our findings were consistent with previous literature on cardiogenic shock in AMI and the increased likelihood of inpatient mortality [20]. AKI is now considered an independent factor when predicting mortality in patients with cardiogenic shock [2]. We found that the AMI inpatients suffering cardiogenic shock are at 17 times more likelihood of inpatient mortality. The emergent nature of cardiogenic shock limits in-depth research on evaluating time-sensitive interventions in this setting. However, this evidence can definitely be used to analyze risk factors by conducting retrospective analysis.

Patients who survive the AKI event may develop CKD and end-stage renal disease, subjecting them to poor quality of life. We saw AKI cohort with AMI inpatients had a significant percentage of patients with major loss of functioning, making them a source of a higher healthcare burden. Hospital-acquired AKI is estimated to cost over ten billion dollars annually [2]. We found the cohort with AKI and AMI inpatients had double the cost of the hospital expense compared to AMI inpatients without AKI. This is an alarming finding and calls for aggressive early identification of patients who are at risk of AKI. In a systematic review, Coca et al. found increased long-term mortality and poor prognosis in patients who survive hospitalization after AKI [21].

Our study findings should be considered with some limitations since it’s a retrospective analysis, and it’s difficult to establish causal relationships. The sample was extracted from the NIS using the diagnostic codes for AMI and AKI and is unable to provide the information on the time frame of these pathologies and their occurrence during the illness course. Secondly, the cardiogenic shock mentioned in the study was identified using the diagnostic codes and the sample was unable to provide clarification regarding whether it led to or occurred following AKI due to the nature of the NIS data. Also, we could not divide the cohort who had undergone percutaneous coronary intervention (PCI) and interventional procedure, which used contrast materials that may have, in turn, increased the risk factor for AKI. This data cannot correlate inpatient mortality rate due to AKI with stages of AKI in AMI inpatients. There may have been underreporting of comorbidities since NIS does not store patient-level clinical information. However, our study strength is our large sample size, and NIS is known for its national representation in terms of the inclusion of population. Also, the study power is increased with a large data set and sample size with minimal reporting bias.

## Conclusions

AMI inpatients with AKI during hospitalization were prevalent in males and whites. Among the demographic risk factors, females and Hispanics had a higher likelihood of in-hospital mortality during the inpatient management of AMI. We found a greater prevalence of comorbidities, longer stay, and higher costs during the hospitalization in the AKI cohort than in the non-AKI cohort. Cardiogenic shock and AKI increased the odds of in-hospital mortality compared to other comorbidities in AMI inpatients. Our findings suggest that the AKI patients had a major loss of functioning due to greater severity of illness compared to the non-AKI cohort. This is an alarming finding and calls for aggressive early identification of patients who are at risk of AKI.
